# Supplementary material for: The genomic landscape of tuberous sclerosis complex
Source: Nat Commun. 2017 Jun 15;8:15816. doi: 10.1038/ncomms15816 (PMC5481739; doi:10.1038/ncomms15816)
Supplement: Supplementary Information — Supplementary figures, supplementary tables, supplementary methods and supplementary references. [file ncomms15816-s1.pdf]

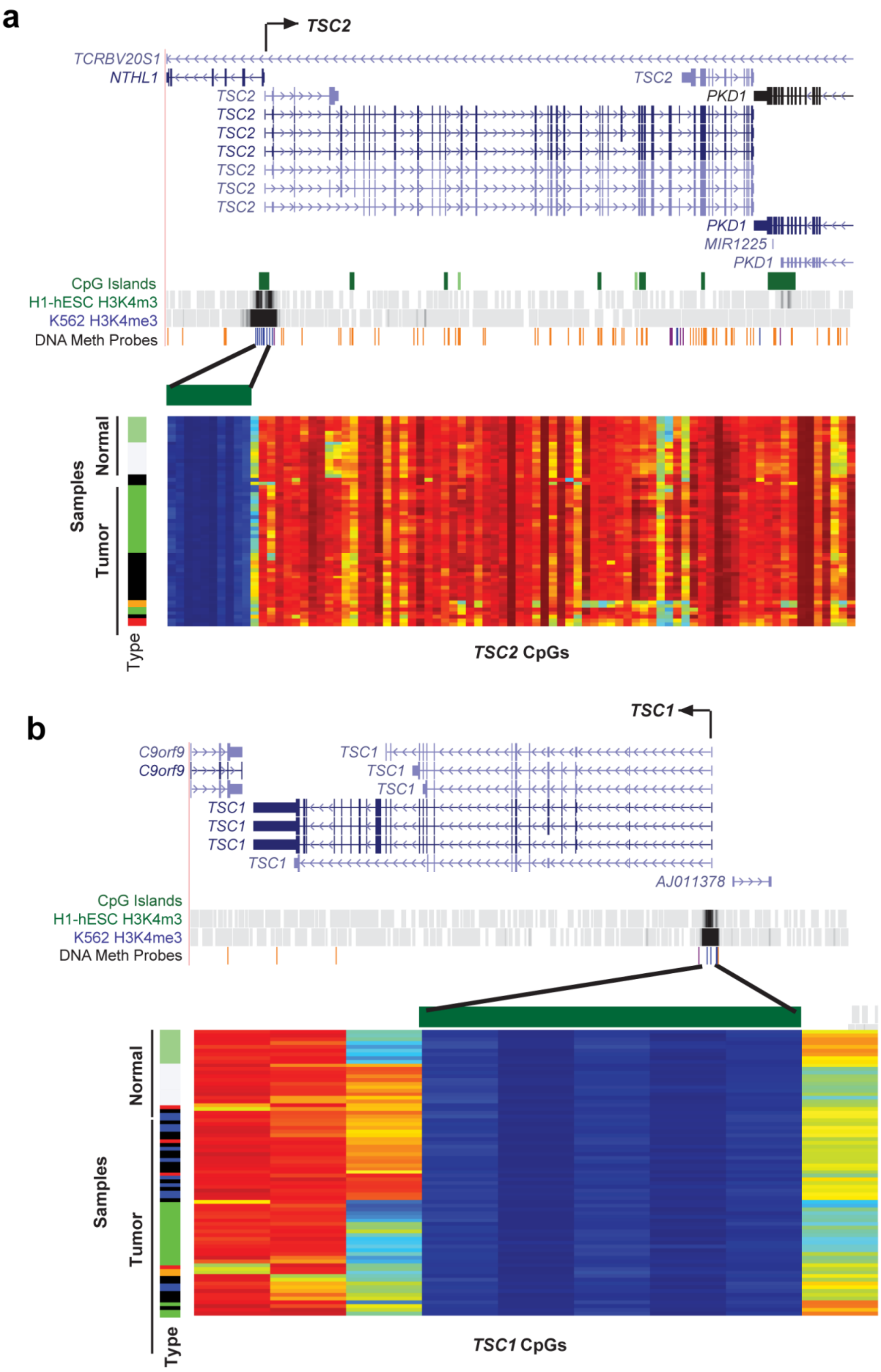

Supplementary Figure 1 TSC1 and TSC2 DNA methylation. (a-b) Methylation of CpG probes along *TSC2* (a) and *TSC1* (b). Transcripts in this region are indicated with blue arrowed lines; CpG island locations are indicated in green; histone Chip-seq (H3K4me3) data are shown in gray. Methylation probe data is shown on a scale from blue (low) to red (high). Tumor type is indicated (light green: normal kidney; gray: normal or matched brain; black: SEGA/SEN: blue: TUB; bright green: RA; red: CRM; orange: skin lesion).

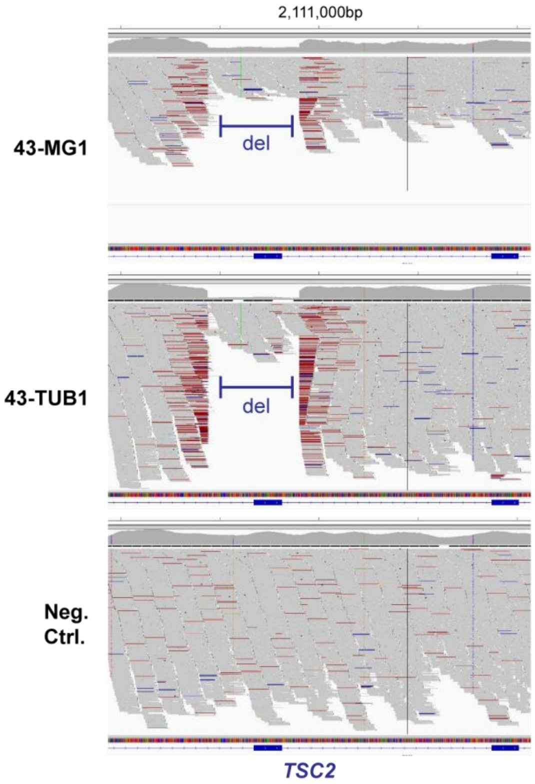

Supplementary Figure 2 Intragenic TSC2 deletion identified by targeted sequencing. A germline deletion (marked by bracket and “del”) of a single exon (blue bar) is observed in patient 43 (43-MG1 blood: top panel; 43-TUB1 cortical tuber: middle panel) as a focal reduction in sequencing reads (gray bars). An unrelated sample is included (bottom panel) for comparison.

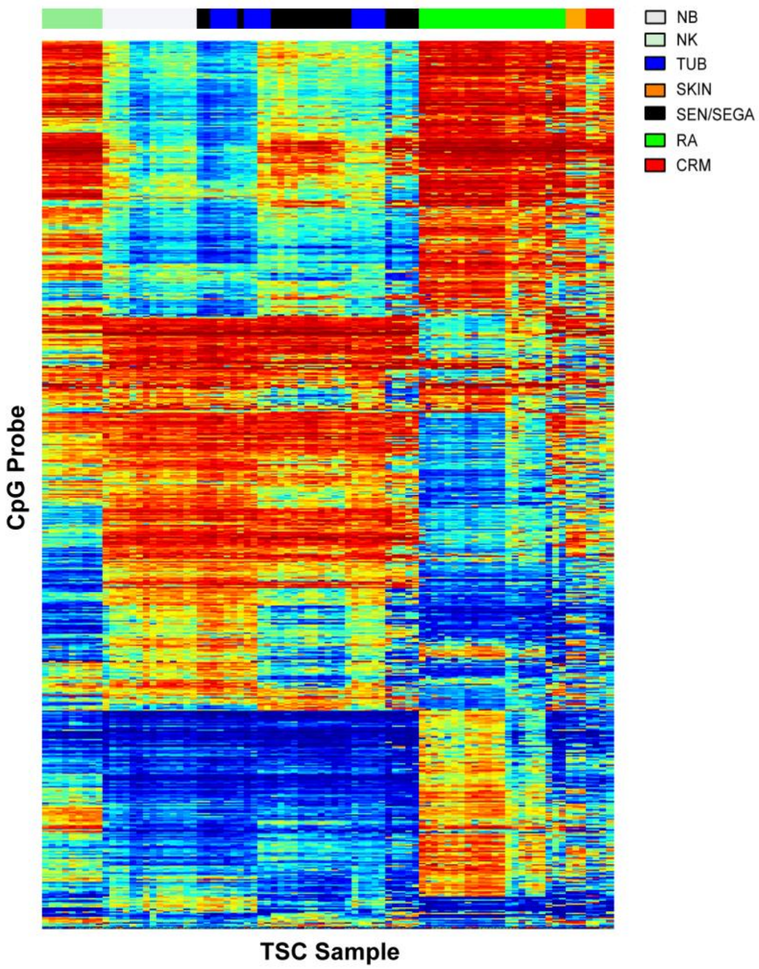

Supplementary Figure 3 DNA methylation profiling of TSC tumors. Unsupervised clustering of CpG probe methylation data in TSC and non-TSC tissues is shown as a heatmap from low (blue) to high (red). Samples are color-coded by tissue type according to the legend.

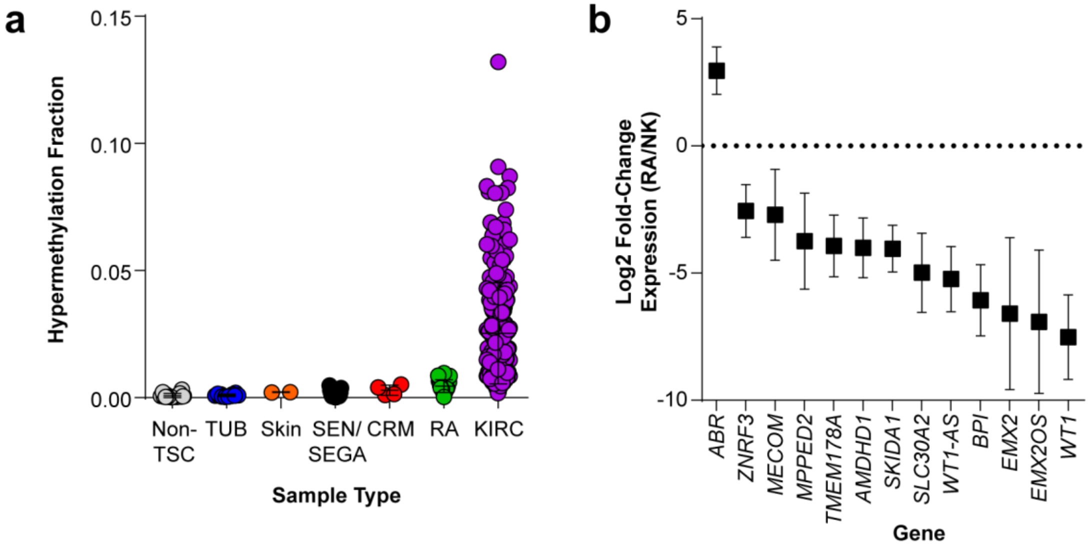

Supplementary Figure 4 DNA methylation features of RAs. A) Hypermethylation fractions of TSC tumors (see Fig. 3a) are shown alongside kidney cancer (KIRC) from the TCGA. (B) The 13 RA-methylated genes with significantly differential expression in RAs versus non-TSC kidney are shown. Square symbols represent mean expression (log2 fold-change in RA versus non-TSC kidney) and bars represent standard deviation.

Supplementary Table 1 FISH validation of CNAs.

Sample	Chr Region	SNP Array Call	FISH Call (if diploid)	FISH Signals	FISH Signals per Cell (% of 200 cells counted)				
					1	2	3	4	6
14-RA1	1p	Loss	Loss	1	76.5%	23.5%	0.0%	0.0%	0.0%
	3q	Loss	Loss	1	76.5%	23.5%	0.0%	0.0%	0.0%
	11p	Loss	Loss	1	76.0%	24.0%	0.0%	0.0%	0.0%
	11q	Gain	Gain	3	0.0%	15.5%	73.5%	0.0%	11.0%
	19p	Loss	Loss	1	75.5%	24.5%	0.0%	0.0%	0.0%
	19q	Loss	Loss	1	75.5%	24.5%	0.0%	0.0%	0.0%
01-RA1	1p	Loss	Loss	1	89.0%	11.0%	0.0%	0.0%	0.0%
	1q	Loss	Loss	1	89.0%	11.0%	0.0%	0.0%	0.0%
01-MK1	1p	Normal	Normal	2	0.0%	100.0%	0.0%	0.0%	0.0%
	1q	Normal	Normal	2	0.0%	100.0%	0.0%	0.0%	0.0%
32-SEGA1	12p	Gain	Gain	3	0.0%	30.0%	63.5%	2.5%	4.0%
	12q	Gain	Gain	3	0.0%	30.0%	63.5%	2.5%	4.0%
29-SEGA1	1p	Loss	Loss	1	92.5%	7.5%	0.0%	0.0%	0.0%
	5p	Gain	Gain	3	0.0%	5.5%	92.5%	0.0%	2.0%
	7p	Loss	Loss	1	91.0%	9.0%	0.0%	0.0%	0.0%
	11p	Loss	Loss	1	91.0%	9.0%	0.0%	0.0%	0.0%
	18p	Loss	Loss	1	89.5%	10.5%	0.0%	0.0%	0.0%
	18q	Loss	Loss	1	89.5%	10.5%	0.0%	0.0%	0.0%
58-CRM1	4p	Gain	Gain	3	0.0%	53.2%	44.0%	0.0%	2.8%
	4q	Gain	Gain	3	0.0%	53.2%	44.0%	0.0%	2.8%
	5p	Gain	N/T	N/T	N/T	N/T	N/T	N/T	N/T
	5q	Gain	N/T	N/T	N/T	N/T	N/T	N/T	N/T
	12p	Gain	Gain	3	0.0%	67.6%	31.2%	0.0%	1.2%
	12q	Gain	Gain	3	0.0%	67.6%	31.2%	0.0%	1.2%
	14p	Gain	N/T	N/T	N/T	N/T	N/T	N/T	N/T
	14q	Gain	Gain	3	0.0%	50.8%	48.0%	0.0%	1.2%
	16p	Gain	Gain	3	0.0%	48.0%	48.8%	0.0%	3.2%
	16q	Gain	Gain	3	0.0%	48.0%	48.8%	0.0%	3.2%
	19p	Gain	Normal	2	0.0%	100.0%	0.0%	0.0%	0.0%
	20p	Gain	N/T	N/T	N/T	N/T	N/T	N/T	N/T
	20q	Gain	N/T	N/T	N/T	N/T	N/T	N/T	N/T
	21p	Loss	N/T	N/T	N/T	N/T	N/T	N/T	N/T
	21q	Loss	Loss	1	48.0%	52.0%	0.0%	0.0%	0.0%
12-RA1	1p	Gain	N/T	N/T	N/T	N/T	N/T	N/T	N/T
38-SEGA1	12p	Gain	N/T	N/T	N/T	N/T	N/T	N/T	N/T
	12q	Gain	N/T	N/T	N/T	N/T	N/T	N/T	N/T
	17p	Gain	N/T	N/T	N/T	N/T	N/T	N/T	N/T
	17q	Gain	N/T	N/T	N/T	N/T	N/T	N/T	N/T
38-SEGA2	12p	Gain	N/T	N/T	N/T	N/T	N/T	N/T	N/T
	12q	Gain	N/T	N/T	N/T	N/T	N/T	N/T	N/T
	17p	Gain	N/T	N/T	N/T	N/T	N/T	N/T	N/T
	17q	Gain	N/T	N/T	N/T	N/T	N/T	N/T	N/T
73-TUB1	7p	Gain	N/T	N/T	N/T	N/T	N/T	N/T	N/T
	7q	Gain	N/T	N/T	N/T	N/T	N/T	N/T	N/T

200 cells counted per sample and number of FISH signals per nuclei reported per probe. Probes chosen based on CNAs identified by SNP array. N/T = not tested. See *Methods.*

Supplementary Table 2 Pathway enrichment of SEGA versus TUB.

GO Biological Process	Total Genes	Matched Genes	Adj. *p* value
INCREASED EXPRESSION			
**Angiogenesis**	231	13	$5.95x10^{-5}$
Positive Regulation of Angiogenesis	104	10	$5.15x10^{-6}$
Receptor-mediated Endocytosis	172	11	$7.26x10^{-5}$
DECREASED EXPRESSION			
**Synaptic Transmission**	432	58	$7.59x10^{-37}$
Neurotransmitter Secretion	65	13	$3.73x10^{-11}$
Synaptic Vesicle Exocytosis	50	10	$6.90x10^{-9}$
**Ion Transport**	321	29	$2.54x10^{-14}$
Ion Transmembrane Transport	291	23	$1.78x10^{-10}$
Regulation of Membrane Potential	127	18	$1.85x10^{-12}$
Neurological System Process	52	12	$4.24x10^{-11}$
Learning	64	11	$5.70x10^{-9}$

Differentially expressed genes (log2 fold-change in SEN/SEGA versus TUB +/- $\geq$ 2 with FDR-adjusted $p \leq 0.001$) were imported into GeneAnalytics (maximum of 300 gene symbols) to identify enriched GO Biological Processes in SEN/SEGA but not cortical tuber (for both increased and decreased expression).

Supplementary Table 3 MetaCore MTORC1 pathway enrichment analysis.

DEG Analysis	Pathway Map	Total	In Data	p-value	FDR-adjusted p-value	Network Objects from Active Data
	Signal transduction_mTORC1 upstream signaling	74	22	0.014	0.027	PDIA3, EGF, ERK1/2, AMPK alpha subunit, IL4RA, Diacylglycerol kinase, zeta, Dsh, HSP90 beta, PLD2, GSK3 beta, MAPKAPK2, WNT, TLR4, IKK-beta, MEK1/2, ERK2 (MAPK1), AKT(PKB), TNF-R1, PDK (PDPK1), p38 MAPK, Axin, Frizzled
SEN/SEGA vs. NB	Signal transduction_mTORC1 downstream signaling	61	18	0.026	0.047	SREBP1 (nuclear), LIPIN1, G6PD, IRS-1, PDCD4, RPS6, Cytochrome c, SREBP2 (nuclear), STAT3, 4E-BP1, p70 S6 kinases, GSK3 beta, eIF4A, ACSL3, MDM2, p53, p70 S6 kinase2, PFKP
	Signal transduction_mTORC1 upstream signaling	74	8	0.033	0.518	EGF, p90RSK1, MAG1, DEPTOR, Dsh, WNT, p90Rsk, Frizzled
RA vs. NK	Signal transduction_mTORC1 downstream signaling	61	6	0.087	0.800	HMGCS2, LIPIN1, SCD, PDCD4, PPAR-gamma, PPAR-alpha
	Signal transduction_mTORC1 upstream signaling	74	3	0.226	0.421	TNF-R1, p38 MAPK, GSK3 beta
TUB vs. NB	Signal transduction_mTORC1 downstream signaling	61	2	0.394	0.572	GSK3 beta, SREBP1 (nuclear)

Differentially expressed genes (log2 fold-change in each lesion type versus normal tissues +/- $\geq$ 1 with FDR-adjusted $p \leq$ 0.001) were imported into MetaCore and algorithm based on the hypergeometric distribution used to calculate enrichment *p*-values. The results from MTORC1-related pathway maps are shown (with FDR adjusted *p*-value less than 0.05 considered significant).

SUPPLEMENTARY METHODS

Whole Exome Sequencing

As necessary, genomic DNA samples were treated with RNAse A (Qiagen) and/or subject to bead purification prior to standard library preparation using a custom (GSL, proprietary) indexing scheme. Exonic DNA was enriched using a SeqCap EZ Human Exome Library v3.0 (NimbleGen) or SureSelect Human All Exon capture kit (Agilent) from genomic DNA. DNA was sonicated to 200 bp in length and then captured according to manufacturers' protocols. After exome protocol completion, libraries were profiled on a BioAnalyzer (or Caliper) for insert size determination and quantitated using Kapa qPCR. Libraries were pooled and clustered at 16-18 pM on the HiSeq 2500 or HiSeq 2000 with high output flowcells and sequenced at 100PE according to Illumina protocols. DNA sequencing was completed at the HudsonAlpha Institute for Biotechnology (HAIB) Genomic Services Laboratory (GSL) or Beijing Genomics Institute (BGI) at the Philadelphia Children's Hospital.

Fastq files were generated using Illumina software and aligned to the hg19 genome with BWA-MEM (v0.7.3a) [http://arxiv.org/abs/1303.3997]. Unmapped reads were removed, SAM files were converted to BAM using SAMTools (v0.1.19), and duplicate reads were removed using Picard MarkDuplicates (v1.88) [http://broadinstitute.github.io/picard/]. BAM files were processed using mitoseek (v1.2) to filter non-uniquely mapping mitochondrial variants. Variant calling was completed using Haplotype Caller in GATK (v3.0) using suggested Best Practices along with the following parameters for SNP filtering (--clusterWindowSize 50 --filterExpression "QD <2.0" --filterName "QD" --filterExpression "FS > 60.0" --filterName "FS" --filterExpression "MQ < 40.0" --filterName "MQ" --filterExpression "HaplotypeScore > 13.0" --filterName "HaplotypeScore" --filterExpression "MappingQualityRankSum < -12.5" --filterName "MappingQualityRankSum" --filterExpression "ReadPosRankSum < -8.0" --filterName "ReadPosRankSum") and (--filterExpression "QD < 2.0" --filterName "QD" --filterExpression "FS > 200.0" --filterName "FS" --filterExpression "ReadPosRankSum < -20.0" --filterName "ReadPosRankSum") for indel filtering[1-3]. Filtered variants were annotated with Variant Effect Predictor (VEP) (v74)[4] and imported to GEMINI[5] to query predicted consequence and genetic relatedness.

Targeted *TSC1/TSC2* Sequencing

For targeted sequencing of TSC1/TSC2, samples enriched using a custom targeted enrichment kit (SeqCap EZ Choice Library, NimbleGen) were multiplexed (9-10 per library hybridization) and sequenced similar to

above at the HAIB GSL using Illumina reagents and the HiSeq 2500. Note, our targeted enrichment kit also includes TBC1D7, although it was not analyzed as part of this study. Quality control and BAM file conversion of the targeted sequencing reads were processed in a similar manner to the WES data. All targeted sequence samples were run in a single cohort using the GATK Haplotype Caller (v3.0) run in GVCF mode (--variant_index_type LINEAR --variant_index_parameter 128000 --emitRefConfidence GVCF) and GenotypeGVCFs was run to merge variant calls. SNPs were filtered using the following parameters (--clusterWindowSize 50 --filterExpression "QD < 2.0" --filterName "QD" --filterExpression "FS > 60.0"--filterName "FS" --filterExpression "MQ < 40.0" --filterName "MQ" --filterExpression "HaplotypeScore > 13.0"--filterName "HaplotypeScore" --filterExpression "MappingQualityRankSum < -12.5" --filterName "MappingQualityRankSum" --filterExpression "ReadPosRankSum < -8.0" --filterName "ReadPosRankSum"). Indel filtering parameters were as follows: ("QD < 2.0" --filterName "QD" --filterExpression "FS > 200.0" --filterName "FS" --filterExpression "ReadPosRankSum < -20.0" --filterName "ReadPosRankSum"). Filtered germline variants were annotated similarly with VEP and GEMINI. In addition, we explored mutations present at low allele frequencies down to 0.5% in the deep sequencing experiment by recalling mutations using LoFreq[6] and VarDict[7]. Source quality was computed using LoFreq with Bonferroni factor 1. Coding consequences of identified mutations are annotated using TransVar[8].We studied missense, nonsense, and mutations affecting splice sites and start codons. For each putative low allele frequency mutation, we required variant allele read support > 3, total read support > 100, and average base quality of variant allele reads > 30. The resulting mutations were manually inspected for artifacts such as strand bias and residual bias from PCR duplication.

TSC1/TSC2 Mutation Calling

The germline or somatic origin of *TSC1/TSC2* mutations in tumors lacking matched non-tumor tissue controls was predicted from features of known germline and somatic mutations as follows. First, all normal tissues had a maximum of one TSC mutation and with the exception of two very low frequency mosaic mutations (74-MG1 and 57-UG1), these were always found at variant allelic fractions (VAF) ≥ 40% when SNVs or INDELs (excluding mosaic mutations: median 49%; range 40% - 72%). These are consistent with heterozygous events affecting an entire population of diploid cells (i.e., germline mutations). Moreover, these mutations detected in

normal tissues were always also detected in tumor tissues from the same patient. Second, CN-LOH events were found exclusively in tumor tissues and always co-occurred with a germline mutation (identified in paired normal tissue). When the germline event was a point mutation, its VAF was always higher in the tumor than paired normal tissue, consistent with CN-LOH leading to enrichment of the mutant allele. Similarly, the VAF of germline mutations in tumors with CN-LOH was higher (median 74%; range 41% - 90%) than the VAF of germline mutations in tumors with all other types of mutations (median 50%; range 34% - 63%). Third, somatic point mutations (those found exclusively in tumor tissue and not in matched normal samples from the same patient) were always found at ≤ 40% VAF (median 17%; range 12% - 40%). Based on this information, we predicted the germline or somatic origin for mutations found in unpaired tumor tissues as follows (predictions are denoted with asterisks in Supplementary Table 2): (*1*) If a tumor had a single mutation present at a VAF > 40%, it was considered a germline event. (*2*) CN-LOH events were always considered somatic, and the additional mutation found in the same tumor was considered the germline event by default (these germline mutations all had VAFs > 40%, as expected; median 69%; range 48% - 90%). (*3*) A point mutation was classified as somatic if it was found at VAF < 40% and occurred with a second mutation at VAF ≥ 40%, which was then classified as germline. If the co-occurring mutation was a large deletion (for which relative frequency could not be clearly determined), they were both classified as "unclear" origin. (*4*) A point mutation was classified as germline if it was found at VAF > 40% and co-occurred with a second mutation at VAF ≤ 40% (if both were ≥ 40% VAF, they were classified as "unclear" origin). (*5*) If a tumor had a single mutation at VAF < 40%, it was classified as "unclear" origin (it could represent a mosaic primary mutation or tumor-specific mutation in the absence of an identifiable primary mutation). Similarly, if a tumor only contained a single large deletion, it was classified as "unclear" origin (as the relative frequency could not be clearly determined). (*6*) If a mutation was observed in two independent tumors (tumors of distinct type) from the same patient, it was considered a germline event.

<u>RNA Sequencing and Differential Gene Expression Analysis</u>

RNA sequencing was completed at the HAIB GSL. Messenger RNA (mRNA) libraries were prepared using NEBNext reagents (New England BioLabs) from total RNA samples. Samples underwent directional sequencing on the Illumina HiSeq 2500 using v4 reagents and 100 bp paired end reads. RNA sequencing read

quality was assessed using FASTQC v0.11.3 (http://www.bioinformatics.bbsrc.ac.uk/projects/fastqc/). Reads were aligned to the hg19 genome using Subread (v1.4.5)[9] with default parameters. Raw read counts to known exons were obtained using FeatureCounts v1.4.5[10] and imported into R[11] for differential expression analysis via limma (v. 3.28.14)[11]. Counts per million (CPM) were calculated and log2 transformed using *voom*[12] followed by trimmed mean of M-values (TMM) normalization via *calcNormFactors* and estimation of the within patient correlations by *duplicateCorrelation*. Low counts were removed based on the number of samples in the smallest group (>1 CPM in at least 2 samples). GeneAnalytics (LifeMap Sciences; geneanalytics.genecards.org) was used for primary gene set enrichment analysis[13]. A maximum of 300 gene symbols were used and up to 10 GO biological processes with medium or high matching scores (FDR-adjusted $p \leq 0.05$) were included in the results. Only processes with at least 10 matched genes were shown in Tables 1 and 2. A follow-up enrichment analysis to search for MTORC1-related signatures was completed using MetaCore. For this, gene-level fold changes and adjusted p-values were imported into MetaCore version 6.29 build 68613 (Thomson Reuters, New York, NY). Pathway analysis was performed using the Pathway Maps One-Click Analysis on genes with an absolute log fold change > 1 and FDR adjusted p-value < 0.001. An algorithm based on the hypergeometric distribution is used to calculate enrichment p-values. Pathway Maps with a FDR adjusted p-value less than 0.05 were considered significant. RNAseq variant calling was conducted using GATK (v3.0) using the suggested Best Practices parameters and with a two-pass STAR (v 2.4.2a) alignment method to the hg19 genome.

CIBERSORT

Publically available RNA sequencing data was downloaded from the NCBI Short Read Archive and used in our CIBERSORT analyses. For RA CIBERSORT analysis, the following datasets were used: adult kidney (SRR2087306, SRR2087307, SRR2087308, SRR2087309, SRR2087325), fetal kidney (SRR643749, SRR643750, SRR643751, SRR643762, SRR643765), adipose tissue (ERR030880, ERR030888, ERR315332, ERR315342, ERR315343, ERR315378, ERR315431, ERR579122, ERR579146), smooth muscle (ERR579125, ERR579131, ERR579153, SRR1617454, SRR1617455, SRR1617456, SRR1617457, SRR1617458, SRR1617459), blood vessel (SRR3192368, SRR3192369), and leukocytes (SRR1747307, SRR1747309, SRR1747311, SRR1747313, SRR1747315, SRR1747317, SRR1747319, SRR1747321,

SRR1747323, SRR1747325). For brain lesions, the following datasets were used: leukocytes (same as above); adult astrocytes (SRR2557093, SRR2557094, SRR2557096, SRR2557097, SRR2557098, SRR2557099, SRR2557100, SRR2557092, SRR2557095), fetal astrocytes (SRR1485144, SRR1485145, SRR2557083, SRR2557084, SRR2557085, SRR2557086, SRR2557087), induced pluripotent stem cell (iPSC) 0 day differentiated neuron (SRR3082572), iPSC 6 day differentiated neuron (SRR3082573), iPSC 18 day differentiated neuron (SRR3082574), iPSC 50 day differentiated neuron (SRR3082575), and whole blood (SRR2443246, SRR2443247, SRR2443248, SRR2443249, SRR2443250, SRR2443251, SRR2443252, SRR2443253, SRR2443254, SRR2443255, SRR2443256, SRR2443257, SRR2443258, SRR2443259, SRR2443260, SRR2443261, SRR2443262, SRR2443263, SRR2537079, SRR2537081, SRR2537082, SRR2537083, SRR2537084). The values for the iPSC neurons were duplicated into two columns to meet CIBERSORT input requirements. Read quality was assessed using FASTQC v. 0.11.3 (http://www.bioinformatics.bbsrc.ac.uk/projects/fastqc/). Reads were aligned to the hg19 genome using Subread (v1.4.5)[9] with default parameters. Raw read counts to known exons were obtained using FeatureCounts v1.4.5[10] and imported into R where counts per million (CPM) were calculated and log2 transformed using *voom* (limma v3.28.7)[12]. For immune cell types, the LM22 gene signature was used[14]. Only samples with estimates yielding p values $\leq$ 0.05 were reported.

SNP Arrays and Copy Number Analysis

Copy number analysis was performed using Infinium HumanOmni2.5S Arrays (Illumina) at the HAIB GSL. From raw IDAT files, the GenomeStudio (v2011.1) Genotyping Module (v1.9) was used to call genotypes and estimate total copy number, log R ratio (LRR), and B-allele frequency (BAF) for each SNP (Illumina). Allele detection and genotype calling were performed using default parameters and the appropriate manifest file (HumanOmni2-5-8-v1-1-C.bpm or HumanOmni25-8v1-2_A1.bpm). For each tumor, total genome-wide copy number estimates were refined using tangent normalization, in which tumor signal intensities are divided by signal intensities from the linear combination of normal samples in the cohort (Tabak B. and Beroukhim R. manuscript in preparation). Individual copy number estimates then underwent segmentation using the Circular Binary Segmentation algorithm[15]. As part of this process of copy number assessment and segmentation, regions corresponding to germline copy number variations (CNVs) were removed by applying filters generated

from germline samples from The Cancer Genome Atlas. Samples with over-segmentation, defined as more than 1000 copy number segments after Circular Binary Segmentation with no enrichment on any particular chromosome, or low data quality were removed from further analysis. Per-sample arm-level and gene-level copy ratios were identified from segmented data using GISTIC 2.0.22[16]. Purity and ploidy estimates and allelic integer copy number (including regions of copy-neutral loss of heterozygosity) were calculated from LRRs and BAFs using ASCAT 2.4[17]. Arm-level copy number events determined by GISTIC 2.0 were visually validated in genome-wide LRR and BAF plots generated by ASCAT 2.4. Chromosomes 9 and 16, as well as the region in chromosome 9q containing *TSC1* and the region in chromosome 16p containing *TSC2*, were visually inspected using genoCN to validate loci with copy-neutral loss of heterozygosity and focal deletions as reported by ASCAT 2.4 and/or GISTIC 2.0[18]. Copy number events detected only visually due to low tumor purity or low signal were also reported.

Array-based DNA Methylation Assay

DNA methylation profiling was completed using Infinium HumanMethylation450 BeadChips (Illumina, San Diego, CA) at the University of Southern California Epigenome Center to obtain DNA methylation profiles. The Infinium HM450 array targets 482,421 CpG sites and covers 99% of RefSeq genes as well as intergenic regions, with an average of 17 CpG sites per gene region distributed across the promoter, 5' UTR, first exon, gene body, and 3' UTR. This platform covers 96% of CpG islands, with additional coverage in island shores and the regions flanking them. We adopted the same pipeline used for The Cancer Genome Atlas (TCGA) project. Namely, we performed bisulfite conversion of genomic DNA from each sample using the EZ-96 DNA Methylation Kit (Zymo Research, Irvine, CA) according to the manufacturer's instructions. We assessed the amount of bisulfite converted DNA and completeness of bisulfite conversion using a panel of MethyLight-based quality control reactions as previously described[19]. Bisulfite-converted DNA was whole genome amplified (WGA) and enzymatically fragmented prior to hybridization to the arrays. BeadArrays were scanned using the Illumina iScan technology, and the IDAT files (Level 1 data) were used to extract the intensities (Level 2 data) and calculate the beta value (Level 3 data) for each probe and sample with the R-based *methylumi* package. Dye-bias normalization and normalization were performed as previously described[20]. The level of DNA methylation at each CpG locus is summarized as avbeta (β) value, calculated as (M/(M+U)) and ranging from 0

to 1, which represents the ratio of the methylated probe intensity to the overall intensity at each CpG locus. A *p* value comparing the intensity of each probe to the background level was calculated with the methylumi package at the same time, and data points with detection *p* values >0.05 were deemed not significantly different from background measurements and therefore were masked as “NA” in the analyses.

<u>Hypermethylation Frequency</u>

To exclude the influence of tissue composition of different tumors, we first identified 126,260 loci consistently unmethylated in 17 distinct normal tissue types relevant to the current study (average β value < 0.3 in all 17 normal tissue types/studies; some studies have higher background level and therefore this threshold is relaxed). The data sources for these normal tissues were as follows: GSE58885 (fetal brain), GSE56515 (fetal adrenal gland, fetal muscle), GSE59157 (fetal kidney and fetal nephrogenic rest), GSE58622 (adult fat), GSE56420 (adult skin), TCGA KIRC project (adult kidney set 1), TCGA KIRP project (adult kidney set 2), GSE53302 (adult muscle), GSE41826 (adult glia, adult neuron), GSE49618 (adult CD34, adult CD3, adult PMN, adult CD19), and unpublished internal data (adult artery). We calculated the hypermethylation frequency as the fraction of these unmethylated loci that had β values > 0.3 for each TSC tumor sample. We also included two kidney cancer datasets from The Cancer Genome Atlas for comparison. For clear cell kidney cancer (KIRC), we used the ‘core+extended’ datafreeze[21] and for papillary kidney cancer (KIRP), we used the full data set[22].

<u>Fluorescent *In Situ* Hybridization (FISH)</u>

FISH probes were prepared from purified BAC clones (BACPAC Resource Center; bacpac.chori.org). The BAC probes used were as follows: RP11-418J17 (1p12); RP1-92O14 (1p34.2; *CDC20*); RP11-74C1, RP11-404E16, RP11-806J18, and RP11-139D23 (1q21.3; *SNX27*); RP11-501M7 (3q24; *SLC9A9*); RP11-326O23 (4p16.2l *STK32B*); RP11-5N8 (5p15.2); RP11-708P5, CTD-2026N22, and RP11-148P17 (7p11.2; *EGFR*); RP11-284G17 and RP11-648P20 (11p13; *WT1*); RP11-151O13 (11p13; *ELP4*); RP11-657B1 and RP11-681H17 (11q13.3; *CCND1*); (12p13.31; *DDX12*); RP11-760G16 and RP11-476D10 (12q12; *LRRK2*); RP11-762M08 (16p13.3; *CASKIN1*); RP11-354N7, RP11-401A12, and RP11-99A10 (16q22.1; *CDH1*); RP11-678G15 (18p11.23; *PTPRM*); RP11-349D12 (18q11.2; *NPC1*); RP11-157B13 (19p12; *ZNF676*); RP11-713I15 (19q13.31; *ZNF226*); RP11-112I22 and RP11-101L2 (21q21.3; *ADAMTS1*). Each clone was labeled with either

Green-dUTP, Orange-dUTP, or Red-dUTP (Abbott Molecular Inc., Abbott Park, IL), by nick translation. Tumor touch preparations were made by imprinting thawed tumors onto positively-charged glass slides. The sample slides were fixed in methanol:acetic acid (3:1) for 30 min, air-dried, aged in 2X saline/sodium citrate (SSC) at 60 °C for 27 min, digested with 0.005% pepsin at 37 °C for 5 min, and washed with 1X PBS for 5 min. Slides were placed in 1% formaldehyde/PBS for 10 min at room temperature, washed with 1X PBS for 5 min, and dehydrated in an ethanol series (70%, 85%, 95%) for 2 min each. Slides were then denatured in 70% formamide/2X SSC at 74 °C for 3.5 min, washed in a cold ethanol series (70%, 85%, 95%) for 2 min each, and air-dried. FISH probes were denatured at 75 °C for 5 min and held at 37 °C for 10-30 min until 10 µl of probe was applied to each sample slide. Coverslips were adhered and slides hybridized overnight at 37 °C in a ThermoBrite hybridization system (Abbott Molecular Inc.). The post-hybridization wash was with 2X SSC at 73 °C for 3 min followed by a brief water rinse. Slides were air-dried and then counterstained with VectaShield mounting medium with 4'-6-diamidino-2-phenylindole (DAPI) (Vector Laboratories Inc., Burlingame, CA). Image acquisition was performed at 600x or 1000x system magnification with a COOL-1300 SpectraCube camera (Applied Spectral Imaging-ASI, Vista, CA) mounted on an Olympus BX43 microscope. Images were analyzed using FISHView v7 software (ASI) and at least 200 interphase nuclei were scored for each sample.

SUPPLEMENTARY REFERENCES

1. DePristo, M.A. *et al.* A framework for variation discovery and genotyping using next-generation DNA sequencing data. *Nat Genet* **43**, 491-8 (2011).
2. McKenna, A. *et al.* The Genome Analysis Toolkit: a MapReduce framework for analyzing next-generation DNA sequencing data. *Genome Res* **20**, 1297-303 (2010).
3. Van der Auwera, G.A. *et al.* From FastQ data to high confidence variant calls: the Genome Analysis Toolkit best practices pipeline. *Curr Protoc Bioinformatics* **43**, 11 10 1-33 (2013).
4. McLaren, W. *et al.* The Ensembl Variant Effect Predictor. *Genome Biol* **17**, 122 (2016).
5. Paila, U., Chapman, B.A., Kirchner, R. & Quinlan, A.R. GEMINI: integrative exploration of genetic variation and genome annotations. *PLoS Comput Biol* **9**, e1003153 (2013).
6. Wilm, A. *et al.* LoFreq: a sequence-quality aware, ultra-sensitive variant caller for uncovering cell-population heterogeneity from high-throughput sequencing datasets. *Nucleic Acids Res* **40**, 11189-201 (2012).
7. Lai, Z. *et al.* VarDict: a novel and versatile variant caller for next-generation sequencing in cancer research. *Nucleic Acids Res* **44**, e108 (2016).
8. Zhou, W. *et al.* TransVar: a multilevel variant annotator for precision genomics. *Nat Methods* **12**, 1002-3 (2015).
9. Liao, Y., Smyth, G.K. & Shi, W. The Subread aligner: fast, accurate and scalable read mapping by seed-and-vote. *Nucleic Acids Res* **41**, e108 (2013).
10. Liao, Y., Smyth, G.K. & Shi, W. featureCounts: an efficient general purpose program for assigning sequence reads to genomic features. *Bioinformatics* **30**, 923-30 (2014).
11. Ritchie, M.E. *et al.* limma powers differential expression analyses for RNA-sequencing and microarray studies. *Nucleic Acids Res* **43**, e47 (2015).
12. Law, C.W., Chen, Y., Shi, W. & Smyth, G.K. voom: Precision weights unlock linear model analysis tools for RNA-seq read counts. *Genome Biol* **15**, R29 (2014).
13. Ben-Ari Fuchs, S. *et al.* GeneAnalytics: An Integrative Gene Set Analysis Tool for Next Generation Sequencing, RNAseq and Microarray Data. *OMICS* **20**, 139-51 (2016).
14. Newman, A.M. *et al.* Robust enumeration of cell subsets from tissue expression profiles. *Nat Methods* **12**, 453-7 (2015).
15. Olshen, A.B., Venkatraman, E.S., Lucito, R. & Wigler, M. Circular binary segmentation for the analysis of array-based DNA copy number data. *Biostatistics* **5**, 557-72 (2004).
16. Mermel, C.H. *et al.* GISTIC2.0 facilitates sensitive and confident localization of the targets of focal somatic copy-number alteration in human cancers. *Genome Biol* **12**, R41 (2011).
17. Van Loo, P. *et al.* Allele-specific copy number analysis of tumors. *Proc Natl Acad Sci U S A* **107**, 16910-5 (2010).
18. Sun, W. *et al.* Integrated study of copy number states and genotype calls using high-density SNP arrays. *Nucleic Acids Res* **37**, 5365-77 (2009).
19. Campan, M., Weisenberger, D., Trinh, B. & Laird, P. MethyLight. *Methods Mol Biol* **507**, 325-37 (2009).
20. Triche, T.J., Weisenberger, D., Van Den Berg, D., Laird, P. & Siegmund, K. Low-level processing of Illumina Infinium DNA Methylation BeadArrays. *Nucleic Acids Res* **41**, e90 (2013).
21. Cancer Genome Atlas Research, N. Comprehensive molecular characterization of clear cell renal cell carcinoma. *Nature* **499**, 43-9 (2013).
22. Cancer Genome Atlas Research, N. *et al.* Comprehensive Molecular Characterization of Papillary Renal-Cell Carcinoma. *N Engl J Med* **374**, 135-45 (2016).
